# Elucidating the Mechanisms of Cell-to-Cell Crosstalk in Probiotics Co-culture: A Proteomics Study of *Limosilactobacillus reuteri* ZJ625 and *Ligilactobacillus salivarius* ZJ614

**DOI:** 10.1007/s12602-023-10133-y

**Published:** 2023-08-15

**Authors:** Iliya Dauda Kwoji, Olayinka Ayobami Aiyegoro, Moses Okpeku, Matthew Adekunle Adeleke

**Affiliations:** 1 Discipline of Genetics, School of Life Sciences, College of Agriculture, Engineering and Sciences, University of KwaZulu-Natal, Westville Campus, Durban, 4000 South Africa; 2 Unit for Environmental Sciences and Management, North-West University, Potchefstroom, Northwest South Africa

**Keywords:** Probiotics, Lactic acid bacteria, Cell-to-cell communication, Quorum sensing, Secretomes

## Abstract

*Limosilactobacillus reuteri* ZJ625 and *Ligilactobacillus salivarius* ZJ614 are potential probiotic bacteria with improved benefits when administered to the host as a multi-strain preparation. To elucidate the mechanisms of cell-to-cell crosstalk between these two strains, we studied their intracellular and extracellular proteomes in co-culture by liquid-chromatography mass-spectrometry (LC-MS) using Dionex Nano-RSLC and fusion mass spectrometer. The experiment consisted of five biological replicates, and samples were collected during the mid-exponential growth phase. The quantitative proteomic profiles revealed several differentially expressed proteins (DEPs), which are down- or up-regulated between and within groups for both the intracellular and extracellular proteomes. These DEPs include proteins synthesising autoinducer-2, a sensor compound for cell-to-cell bacterial crosstalk during quorum sensing in mixed culture. Other important DEPs identified include enolase, phosphoglycerate kinase, and l-lactate dehydrogenase, which play roles in carbohydrate metabolism. Proteins associated with transcription, ATP production and transport across the membrane, DNA repair, and those with the potential to bind to the host epithelium were also identified. The post-translational modifications associated with the proteins include oxidation, deamidation, and ammonia loss. Importantly, this study revealed a significant expression of S-ribosylhomocysteine lyase (*lux*S) involved in synthesising autoinducer-2 that plays important roles in quorum sensing, aiding bacterial cell-to-cell crosstalk in co-cultures. The proteome of *L. salivarius* ZJ614 was most affected when co-cultured with *L. reuteri* ZJ625. In contrast, omitting some medium components from the defined medium exerted more effects on *L. reuteri* ZJ625 than *L. salivarius* ZJ614.

**Supplementary Information:**

The online version contains supplementary material available at 10.1007/s12602-023-10133-y.

## Introduction

Probiotics are safe-to-consume microbial preparations that modulate the gut microbiome and enhance mucosal immunity through interactions with the host intestinal epithelium [1]. Although the mechanism of actions of probiotics is not fully understood, these beneficial microbes are characterised by some molecular effectors on the cell that interacts with the gut microbiome and produces bioactive substances [2]. Probiotics alter the gastric pH, compete with pathogenic microbes for nutrients and attachment sites, and inhibit the proliferation of pathogenic organisms by producing antimicrobial substances such as bacteriocins [3–5]. Probiotic-microbiome interactions contribute to health by suppressing the proliferation of pathogens, particularly in oral or vaginal microbial imbalances [5]. The intake of probiotics re-establish gut microbiome balances, maintains intestinal barrier integrity, modulates immune responses, inhibits pathogens, and ameliorates chronic inflammation [6].

Some microbes with probiotic effects include species and strains of yeast, lactobacilli, and *Bifidobacterium* commonly used in food, drugs, and feed additives [7]. Traditionally, *Bifidobacterium* and lactobacilli are abundant in the gut and fermented foods as starter cultures during fermentation [2, 8, 9]. Recently, other bacterial species, including *Akkermansia muciniphila* [10] and *Faecalibacterium prausnitzii* have been identified to benefit the host and termed next-generation probiotics [11]. Maintaining the gut microbiome and its interactions with the host is important in sustaining intestinal homeostasis [12]. Clinical studies have revealed the potential of probiotics to ameliorate acute inflammation in pouchitis patients [13–15]. Probiotics also improve the absorption of amino acids in the gut and enhance the growth performance of mammals post-weaning [16, 17]. LAB inhibits food spoilage bacteria and prolongs the shelf life of fermented food [18]. *Lactobacillus* and *Bifidobacterium*, including *L. acidophilus*, *Bifidobacterium longum*, *Ligilactobacillus salivarius*, and *Limosilactobacillus reuteri*, regulate the levels of inflammatory cytokines such as TNF-α, IL-6, and IL-1β [19]. Despite this, knowledge of the mechanisms of host-microbe interactions and microbial structures relating to probiotic actions are poorly understood [20].

*Limosilactobacillus reuteri* is an ideal organism for studying the evolutionary behaviour of gut symbionts primarily due to its ability to colonise diverse hosts, including birds [21]. In some vertebrates like pigs, chickens, and rodents, *L. reuteri* is a dominant species that forms a biofilm-like union with the epithelium of the upper digestive tract [22, 23] and modulates the gut microbiome [24, 25]. *L. reuteri*, like other probiotics, exerts anti-inflammatory effects, enhances gut barrier functions, stimulates the repair of damaged mucosa [26, 27], and inhibits pathogens through the secretion of organic acids, ethanol, reuterin, and reutericyclin [28]. Furthermore, *L. reuteri* maintains health and alleviates disease along the gut-brain axis [29]; attenuates hepatic diseases [30], inflammatory bowel disease, cystic fibrosis [31]; and enhances insulin sensitivity [32]. *Ligilactobacillus salivarius* also play functional roles in strengthening the intestinal integrity [33] and decreasing inflammatory cytokines in the serum and bacterial translocations and modulates the gut microbiome [34]. Feeding piglets with *L. salivarius* post-weaning increases daily weight gain while alleviating post-weaning diarrhoea [24, 35]. Thus indicating the potential of *L. salivarius* to prevent and mitigate colonic inflammation by modulating the gut microbiota [36]. To fully derive probiotics benefits, understanding strain-specific actions is necessary for a targeted therapeutic approach development [12].

Proteomics is the study of the identities and quantification, prediction of complexes, interactions, post-translational modifications, and cellular localization of the proteins expressed in a cell or an entire organism [37, 38]. Additionally, proteomics is used to study the responses of a biological system to defined physiological perturbations [38]. The ability of probiotics to attach to the host intestine is partly mediated through the presence of surface proteins [39]. Hence, studying the intracellular and surface proteome (secretome) of *L. reuteri* ZJ625 and *L. salivarius* ZJ614 in co-culture can reveal the mechanism of intercellular interactions between the two strains. To elucidate the mechanisms of intercellular crosstalk between *L. salivarius* ZJ614 and *L. reuteri* ZJ625 in co-culture, proteomics was employed to study the differentially expressed proteins and their associations with different physiological processes. We also examined the post-translational modifications and protein-protein interactions to understand how co-culturing affects the intracellular and extracellular proteome of *L. salivarous* ZJ614 and *L. reuteri* ZJ625.

## Methodology

### Bacterial Source

The isolates were sourced from the culture collection of the Gastrointestinal Microbiology and Biotechnology Unit, Agricultural Research Council, Animal Production Institute Irene, Pretoria, South Africa, based on a previous study where *L. salivarius* ZJ614 and *L. reuteri* ZJ625 were isolated from indigenous South African Windsnyer pigs. *L. salivarius* ZJ614 and *L. reuteri* ZJ625 were characterised and confirmed for probiotics properties such as auto- and co-aggregation, resistance to gastric acid, and the potential to transfer antimicrobial resistance genes. When tested in experimental animals, they performed better when administered in a co-culture [24].

### Experimental Design

The nutritional needs of *L. salivarius* ZJ614 and *L. reuteri* ZJ625 were determined to formulate defined media that adequately support their growth. The minimal defined media was prepared following a series of leave-one-out experiments to determine the minimum nutrient requirements of the strains. Moreover, we examined the growth kinetics of the strains in these media to establish the optimal sampling points to harvest them for proteomics studies. The results of the composition of the defined media and the growth behaviours of *L. salivarius* ZJ614 and *L. reuteri* ZJ625 were published in our previous work [40]. The experiment was performed by culturing *L. salivarius* ZJ614 and *L. reuteri* ZJ625 in single- and co-cultures in the complete chemically defined (CDM) and minimally defined (MDM) media with five biological replicates resulting in fifteen CDM and MDM cultures, respectively, and a total of thirty (30) samples for both. The culture of *L. salivarius* ZJ614 in CDM and MDM is abbreviated CLS and MLS, respectively. Of *L. reuteri* ZJ625, CDM is CLR, and MLR is for culture in MDM. For the co-cultures of both isolates, the culture in MDM is abbreviated as MLRLS, while for CDM, CLRLS was used.

### Culture and Sample Preparations

The strains were cultured singly and in co-cultures anaerobically at 37 °C overnight. The bacterial growth was arrested at 18 h post-incubation, corresponding to the mid-exponential growth phase. The harvested cultures were centrifuged (12,000 × g) for 30 s, and the cell pellets were washed twice using 0.8% physiological buffered saline solution and straightway stored at − 80 °C. The supernatant (for secretome analysis) was collected in sterile 2-ml Eppendorf tubes for the respective samples and stored at − 80 °C.

### Extraction of Intracellular and Extracellular Proteins

The bacterial cell pellets were resuspended in Tris-buffer having 5 mM Tris (2-carboxyethyl) phosphine hydrochloride (TCEP) and placed in a cooled sonic batch for 30 s, followed by vortex at high speed for 30 s. This procedure was repeated 5 times for each sample, followed by centrifuging (at 12,000 × g) the solubilised pellets for 10 min to pellet insoluble material. Protein concentration was then determined from the soluble portion.

Secretome samples (medium supernatant) were concentrated using 4 mL Amicon 3 kDa NMW cut-off filters. The samples were concentrated to 500 mL before dilution with Tris buffer to 5 mL (10 × dilution). This was repeated twice before protein concentration was determined at 280 nm using a NanoDrop spectrophotometer (Thermo Scientific).

### On-Bead Protein Digestion

The chemicals used for this work are analytical grade or equivalent. The extracted proteins were diluted in 100 mM Tris–HCl (Sigma) containing 100 mM NaCl and 1% SDS (Sigma) followed by reduction using 5 mM triscarboxyethyl phosphine (TCEP; Fluka) in 100 mM Tris buffer at 60 °C for 1 h. Methylation of cysteine residues was carried out using 20 mM S-methyl methanethiosulfonate (Sigma) in 100 mM triethylammonium bicarbonate (TEAB) at room temperature for 30 min. Following thiol methylation, a solution of 100 mM ammonium acetate, 30% acetonitrile, and pH 4.5 was used as a binding buffer to dilute the samples twofold. The protein solution was then added to MagResyn (Resyn Biosciences), a hydrophilic interaction liquid chromatography (HILIC) magnetic particle prepared according to the manufacturer’s guide and incubated at 4 °C overnight to allow for protein binding. This was followed by removing the supernatant and washing the magnetic particles using the washing buffer (95% acetonitrile (ACN; Romil) twice. The washing was proceeded by suspending the magnetic particles in 25 mM ammonium bicarbonate containing trypsin (New England Biosystems) at a ratio of 1:50. The mixture was then incubated for 18 h at 37 °C, followed by peptide extraction using 50 mL of 15-trifluoroacetic acid (TFA) (Sigma). The samples were lyophilised and dissolved in 50 mL 2% acetonitrile-H_2_O; 0.1% formic acid solution (FA; Sigma) for analysis.

### Liquid-Chromatography Mass-Spectrometry Analysis

Liquid chromatography was performed using the Dionex nano-RSLC system with a 5 mm × 300 μm trap column (Thermo Scientific) and a CSH 25 cm × 75 μm × 1.7 μm particle size analytical column. The solvent system used consisted of loading-solvent A (2% acetonitrile–water; 0.1% formic acid) and solvent B (100% acetonitrile–water). The samples were loaded onto the trap column using loading solvent at a flow rate of 2μL/min from a temperature-controlled autosampler set at 7 °C. Loading was performed for 5 min before the sample was eluted onto the analytical column. The flow rate was set to 300 nL/minute, and the gradient generated 5.0–30%B over 60 min and 30–50%B from 60 to 80 min. Chromatography was performed at 45 °C, and the outflow was delivered to the mass spectrometer.

A fusion mass spectrometer fitted with a NanoSpray Flex ionisation source (Thermo Scientific) was used for mass spectrometry analysis. The samples were infused via a stainless-steel nano-pore emitter. A positive mode with a spray voltage of 1.8 kV and ion transfer capillary at 275 °C was used for data collection. Internal spectral standardisation was performed using polysiloxane ions with a mass-to-charge ratio (m/z) of 445.12003. An orbitrap detector was used in acquiring MS1 scans and data in profile mode. The orbitrap detector was programmed to scan at 120,000 resolutions over a range of 375 to 1500 with an automatic gain control (AGC) target of 4 E5 and the highest injection time of 50 ms. To acquire MS2, a monoisotopic precursor selection of the ion with charges + 2 to + 7 and an error tolerance of ± 10 ppm was used. Precursor ions were excluded from fragmentation once for a period of 60 s. The quadrupole mass analyser with an HCD energy of 30% was employed for selecting the precursor ions for fragmentation in HCD mode. The data were obtained in centroid mode following fragment ions detection in the Orbitrap mass analyser at 30,000 resolutions and AGC target of 5E4 at the highest injection time of 100 ms.

### Bioinformatics and Data Analysis

The raw data generated from the mass spectrometer were imported into Proteome Discoverer v1.4 (Thermo Scientific) and processed using the Sequest algorithm. The processed data were parsed against the Lactobacilli database using a semi-tryptic cleavage with 2 allowed missed cleavages. The precursor and fragment mass tolerances were 10 ppm and 0.02 Da, respectively. Deamidation (NQ) and oxidation (M) were set as dynamic modifications. Peptide validation was performed using the target-decoy PSM validator node. The search results were imported into Scaffold Q + (v5.2.2) (www.proteomesoftware.com) for downstream statistical analysis. Student’s *t*-test (*p* < 0.05, Benjamini-Hochberg) employing multiple test corrections and normalisation of 0.0 was carried out on the data to determine the statistical significance of the expressed proteins and identification of differentially expressed proteins (DEPs). The DEPs and their levels of expression, *p*-values, and functions were presented as tables. Furthermore, protein-protein interaction (PPI) network analyses were carried out using String Version 11.5 (https://string-db.org/) [41]. The PPI data were exported as vector files for network analyses using Cytoscape v3.9.1 (https://apps.cytoscape.org/) [42] and annotation/interpretation using AutoAnnotate v1.4.0 (http://baderlab.org/Software/AutoAnnotate), a Cytoscape sub-software for network analysis and interpretation [43]. The functions of the DEPs were determined by mapping the identified protein on the UniProt database via the UniProtKB using ChEBI (https://www.uniprot.org/) [44], and pathways analyses using Kyoto Encyclopedia of Genes and Genomes (KEGG) (https://www.genome.jp/kegg/pathway.html). The functional enrichment data were exported in tab-delimited formats and summarised by FDR for visualisation using Tidyverse v1.3.0 [45] in R v4.2.1 [46].

### Criteria for Protein Identification

The MS/MS-based peptide and identified proteins were confirmed using Scaffold (version 5.1.2, Proteome Software Inc., Portland, OR). Identified peptides were accepted at more than 88.0% probability of achieving a false discovery rate (FDR) of less than 0.1%. The Scaffold Local FDR algorithm was used to assign the peptide probabilities from Sequest. In contrast, the peptide prophet algorithm set the peptide probabilities from X! Tandem [47] with Scaffold delta-mass correction. Proteins were identified at a greater than 6.0% probability of obtaining an FDR of less than 1.0% and having not less than 2 identified peptides. Protein probabilities were assigned by the Protein Prophet algorithm [48]. However, proteins containing similar peptides, not differentiable based on MS/MS analysis alone, were classified to satisfy the principles of parsimony. In contrast, the proteins having significant peptide evidence were grouped into clusters.

## Results

### Bacterial Growth and Cell Density

The strains were harvested from the defined media at 18 h post-incubation after measuring the pH and growth densities at 600 nm. The pH of the bacterial culture indicates a drop in pH 18 h post-incubation for the experimental groups, irrespective of culture conditions. The growth densities indicate relatively higher densities in the co-culture compared to the single cultures on both the complete and minimal defined media. The average growth densities and pH for the growth of *L. reuteri* ZJ625 and *L. salivarius* ZJ614 were computed and presented in Table 1.

**Table 1:** The average growth densities and pH of the growth of *L. reuteri* ZJ625 and *L. salivarius* ZJ614 and their co-culture in defined media

**Medium**			***L. salivarius***			***L. reuteri***			***L. reuteri*** + ***L. salivarius***		
	**pH**	**OD**_**600**_	**OD**_**600**_		**pH**	**OD**_**600**_		**pH**	**OD**_**600**_		**pH**
			0 h	18 h	18 h	0 h	18 h	18 h	0 h	18 h	18 h
**CDM**	6.14	0.012	0.068	4.70	4.06	0.064	4.89	4.50	0.068	5.96	3.41
**MDM**	6.14	0.013	0.058	4.12	3.96	0.054	4.74	4.38	0.062	5.68	4.02

*CDM* complete defined media, *MDM* minimal defined media, *OD*_*600*_ optical density at 600 nm, *h* incubation time in hours

### Protein Identification

**Fig. 1:**
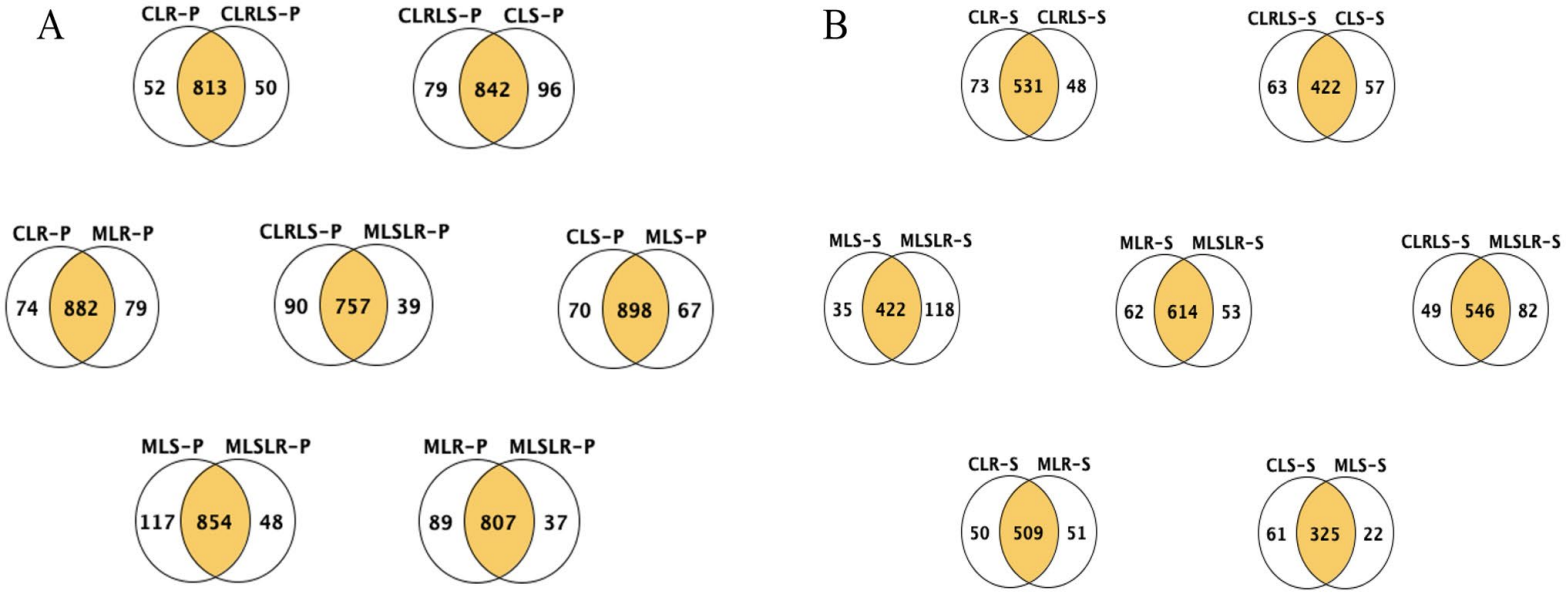
Venn diagrams summary of the total and unique proteins identified between and within groups of the growths of *L. salivarius* ZJ614 and *L. reuteri* ZJ625 in defined media. **A** Intracellular proteins and **B** extracellular proteins. Key: CLR-P and CLS-P: *L. reuteri* ZJ625 and *L. salivarius* ZJ614 proteomes, respectively, when cultured in a complete defined medium. MLR-P and MLS-P; *L. reuteri* ZJ625 and *L. salivarius* ZJ614 proteomes, respectively, when cultured in a minimal defined medium (MDM). CLRLS-P and MLRLS-P; co-cultures of *L. reuteri* ZJ625 and *L. salivarius* ZJ614 proteomes in complete and minimal defined media

The samples were compared between groups to identify the proteins and differential proteins from the different experimental groups. The proteins were identified at *p* < 0.05, 1.0% false discovery rate (FDR) protein threshold, minimum peptides of 2 and peptide threshold of 0.1% FDR. For the group comparison of the intracellularly expressed proteins (Fig. 1A), the highest total amount of proteins was observed from *L. reuteri* ZJ625 in CDM and MDM (CLR vs CLR) and that of *L. salivarius* ZJ614 in CDM and MDM (MLS vs CLS), while the lowest number of proteins were observed between the co-cultures in the complete and minimal defined media (CLRLS vs MLRLS). For the extracellularly expressed proteins (secretome), the highest number of proteins was observed between *L. reuteri* ZJ625 and the co-culture (CLR vs CLRLS). In contrast, the lowest was observed in CLS vs MLS. The numbers of unique and common proteins expressed between and within groups for intracellularly and extracellularly expressed proteins are presented in Fig. 1A, B respectively.

### Identification of Differentially Expressed Proteins (DEPs)

To identify the DEPs between the groups, quantitative protein profiles revealed the expression level (either high or low) using a student *t*-test at *p* < 0.05. The analysis of the DEPs of the intracellularly expressed proteins between CLR and CLRLS showed two significant differential proteins linked to the 50 s ribosomal protein, which is high in the co-culture (*L. reuteri* ZJ625 plus *L. salivarius* ZJ614) and low in the single culture. Of the total expressed proteins between CLS and CLRLS, 420 proteins were significantly differentially expressed. The DEPs include clusters of elongation factor TU, Chaperon GroEl, DNA-directed RNA polymerase subunit, 30S ribosomal proteins, ATP synthase subunits, and isoleucine tRNA ligase. The comparison of MLRLS and CLRLS revealed 149 DEPs that are either up- or down-regulated depending on the experimental group. The differential proteins observed from MLRLS vs CLRLS include S-ribosylhomocysteine lyase (*lux*S), phosphocarrier protein HPr, Redox-sensing transcriptional repressor *Rex*, chaperon GroEL, a cluster of protein translocase subunit, and chaperon protein DnaK, among others. Comparison of CLR and MLR revealed 47 differential proteins (*p* < 0.05), which are also associated with chaperone GroEL, a cluster of arginine tRNA ligase, ornithine carbamoyl transferase, and a cluster of phosphoglycerate kinase. Although differential proteins were observed when CLS and MLS were compared, the quantitative profile was insignificant between these groups. Also, no significant DEPs were identified between MLR and MLRLS, and only one DEP belonging to a cluster of aspartate tRNA ligase was observed between MLS and MLRLS (Table 2).

**Table 2:** Intracellular proteome: differentially expressed proteins between and within groups for the culture of *L. salivarius* ZJ614 and *L. reuteri* ZJ625 in defined media

**Groups**	**Proteins (genes)**	**Quantitative profile**		**p-value**	**Functions/associated pathways**
		**Up regulated**	**Down regulated**		
**CLR vs CLRLS**	50S ribosomal protein (*rpl*B)	CLRLS	CLR	< 0.00010	Enhance the function of the aminoacyl-tRNA binding site
**CLS vs CLRLS**	L-lactate dehydrogenase (*ldh*)	CLS	CLRLS	0.0013	Catalyses the conversion of lactate to pyruvate
	Chaperonin GroEL	CLS	CLRLS	0.0077	Together with its co-chaperonin GroES, plays an important role aiding protein folding
	Phospho-glucosamine mutase (*glm*M)	CLRLS	CLS	0.0011	Enzymatically regulates the formation of glucosamine-1-phosphate from glucosamine-6-phosphate
	Amidotransferase subunit B (*gat*B)	CLS	CLRLS	< 0.00010	Regulates the ATP-dependent conversion of formylglycinamide ribonucleotide and glutamine to yield formylglycinamidine ribonucleotide and glutamate
	Elongation factor Tu (*tuf*)	CLS	CLRLS	0.00026	Induces the exchange of GDP to GTP
	GMP synthase (*gua*A)	CLRLS	CLS	0.0041	Regulates the production of GMP from XMP
	DNA mismatch repair protein (*Mut*L)	CLS	CLRLS	0.014	Functions in repairing mismatches in DNA
	GTPase (*der*)	CLRLS	CLS	0.0010	Regulates bacterial responses to stress, cell cycle control, ribosome biogenesis and structural formation
	GMP reductase (*gua*C)	CLS	CLRLS	0.010	Catalyses the alteration of nucleoside and nucleotide derivatives of G to A, and in maintaining the balances of A and G nucleobases within the cell
	Phosphoglycerate kinase (pgk)	CLS	CLRLS	0.0033	Regulates carbohydrate breakdown during glycolysis; and pyruvate production from D-glyceraldehyde 3-phosphate
	Enolase (*eno*)	CLS	CLRLS	0.014	Functions in enzymatic breakdown of carbohydrate during glycolysis and the reversible alteration of 2-phosphoglycerate into phosphoenolpyruvate
	Glucosamine-6-phosphate deaminase (*glm*M)	CLS	CLRLS	0.00021	Catalyses the reversible formation of fructose 6-phosphate (Fru6P) and ammonium ion through the reversible isomerization-deamination of glucosamine 6-phosphate (GlcN6P)
	Pyrrolidone-carboxylate peptidase (*pcp*)	CLS	CLRLS	0.00041	Release of an N-terminal pyroglutamyl group from a polypeptide
	Dihydrofolate reductase (*fol*A)	CLS	CLRLS	0.0057	Regulates folate metabolism and de novo production of glycine, purine, and DNA precursors
	HPr kinase/phosphorylase (*hpr*K)	CLS	CLRLS	0.0019	Catalyses phosphorolysis of seryl-phosphorylated HPr (P-Ser-HPr) and acts as a sensor enzyme that regulates carbon metabolism and sugar transport
	N-acetylglucosaminyl transferase (*gtf*3)	CLRLS	CLS	< 0.00010	Catalyses the transfer of N-acetylglucosamine to the terminal portion of the 3-O-(N-acetyl-α-D-glucosaminyl)-L-seryl- resulting from the first step of glycosylation during the second phase
	DNA polymerase III PolC-type (*pol*C)	CLRLS	CLS	0.0080	Needed for replication during DNA synthesis
	Elongation factor G (*fus*A)	CLRLS	CLS	< 0.00010	Catalyses the GTP-dependent ribosomal translocation step during translation elongation
	Glyceraldehyde-3-phosphate dehydrogenase (GAPDH) (*gap*)	CLS	CLRLS	0.0061	Carbohydrate metabolism; glycolysis; produce pyruvate from D-glyceraldehyde 3-phosphate
**CLRLS vs MLRLS**	Protein translocase subunit *Sec*A	MLSLR	CLRLS	0.0036	Facilitate the translocation of polypeptide chains across the membrane
	DNA-directed RNA polymerase subunit beta (*rpo*B)	MLSLR	CLRLS	0.0060	Acts on the ribonucleic acid bases triphosphates to facilitate the transcription of DNA into RNA
	ATP synthase subunit-β (*atp*D)	CLRLS	MLSLR	0.00016	Plays a role in ATP synthesis from ADP across the cell membrane
	Chaperone protein (*dna*K)	CLRLS	MLSLR	< 0.00010	Prevents the accumulation of stress-denatured proteins in an autonomous Dnak-independent way to avert the effects hyperosmotic and heat shock
	Glucose-6-phosphate isomerase (*pgi*)	CLRLS	MLSLR	0.0043	Catalyses the reversible isomerization of glucose-6-phosphate to fructose-6-phosphate
	S-ribosylhomocysteine lyase (*lux*S)	MLSLR	CLRLS	< 0.00010	Involved in bacterial intercellular crosstalk of cell density and metabolic potential of the environment through the synthesis of autoinducer 2 (AI-2). Also regulates the transformation of S-ribosylhomocysteine to homocysteine and 4,5-dihydroxy-2,3-pentadione
	Redox-sensing transcriptional repressor (Rex)	MLSLR	CLRLS	0.00046	Modulates transcription during changes in the cellular NADH/NAD^+^ redox state
	Elongation factor Ts (*tsf*)	MLSLR	CLRLS	0.0013	Promotes binding of aminoacyl-tRNA to the ribosomes during protein biosynthesis
	Phosphocarrier protein HPr	MLSLR	CLRLS		Regulates the phosphorylation of incoming sugar substrates and their translocation across the cell membrane. It acts as a carbohydrate active-transport system
	Phosphopentomutase (*deo*B)	MLSLR	CLRLS	0.00051	Catalyse phosphotransfer between the C1 and C5 carbon atoms of pentose
	Exodeoxyribonuclease 7 large subunit (*xse*A)	MLSLR	CLRLS	< 0.00010	Bidirectionally degrades single-stranded DNA into large acid-insoluble oligonucleotides, which are then degraded further into small acid-soluble oligonucleotides
	S-adenosylmethionine synthase (*met*K)	CLRLS	MLSLR	0.00034	Plays role in amino acid biosynthesis in catalyses the formation of S-adenosylmethionine from methionine and ATP
	Protein GrpE (*grp*E)	CLRLS	MLSLR	0.0021	acts in bacterial response to hyperosmotic and heat shock by preventing the accumulation of stress-denatured proteins, in association with DnaK and GrpE
	4-hydroxy-tetrahydrodipicolinate synthase (*dap*A)	MLSLR	CLRLS	< 0.00010	Enzymatically influence the condensation of (S)-aspartate-beta-semialdehyde [(S)-ASA] and pyruvate to 4-hydroxy-tetrahydrodipicolinate (HTPA) (amino acid biosynthesis)
	Ribose-5-phosphate isomerase A (*rpi*A)	CLRLS	MLSLR	0.00039	Metabolism of carbohydrate and pentose-phosphate pathway catalyses the reversible conversion of ribose-5-phosphate to ribulose 5-phosphate
**CLR vs MLR**	Ornithine carbamoyl transferase (*arc*B)	CLR	MLR	0.00036	Enzymatically regulates the transfer of carbamoyl group from carbamoyl phosphate to the N atom of ornithine for the production L-citrulline
	Arginine deiminase (*arc*A)	CLR	MLR	0.00083	Facilitates the breakdown of Amino-acid (L-arginine) via ADI pathway and carbamoyl phosphate from L-arginine:
	Trigger factor (*tig*)	CLR	MLR	0.00016	Acts as a molecular chaperone during protein folding and maintenance of freshly produced proteins in open conformation
	Adenylate kinase (*adk*)	CLR	MLR	0.00013	Regulates adenine nucleotide metabolism and maintains cellular energy homeostasis
	Serine–tRNA ligase	CLR	MLR	0.00013	Binds serine to tRNA
	Argininosuccinate synthase	CLR	MLR	0.00072	Regulates the biosynthesis of L-arginine from L-ornithine and carbamoyl phosphate
	Galactokinase (*gal*K)	CLR	MLR	0.0014	Regulates the transfer of the ATP γ-phosphate to D-galactose during the formation of α-D-galactose-1-phosphate
	Carbamoyl-phosphate synthase large chain	CLR	MLR	0.00022	Regulates amino-acid and pyrimidine metabolism
	Bifunctional protein FolD	CLR	MLR	< 0.00010	Catalyses the oxidation and subsequent hydrolysis of 5,10-methylenetetrahydrofolate 10-formyltetrahydrofolate
	ATP-dependent zinc metalloprotease	MLR	CLR	0.0012	Control the quality of integral membrane proteins
	NAD(P)H-hydrate epimerase	CLR	MLR	0.00088	Epimerize a damaged NAD(P)H resulting from enzymatic or heat-dependent hydration to aid in repair
	Global transcriptional regulator Spx	CLR	MLR	0.00048	Functions in stress response through a positive or negative genes regulation
**MLSLR vs MLS**	Cluster of Aspartate–tRNA ligase	MLSLR	MLS	< 0.00010	Binds L-aspartate to tRNA (Asp)
	Peptide chain release factor 1 (*prf*A)	MLSLR	MLS	< 0.00010	Peptide chain release factor 1 directs the termination of translation in response to the peptide chain termination codons UAG and UAA
	Ribosome maturation factor (RimM)	MLSLR	MLS	0.00028	An accessory protein needed during the final step in the assembly of 30S ribosomal subunit, possibly for assembly of the head region. Probably interacts with S19. Essential for efficient processing of 16S rRNA. May be needed both before and after RbfA during the maturation of 16S rRNA. It has affinity for free ribosomal 30S subunits but not for 70S ribosomes

**Fig. 2:**
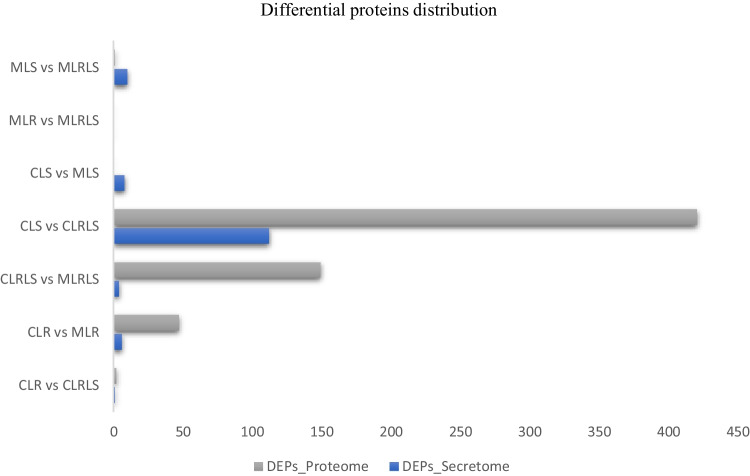
Distribution of differentially expressed proteins between and within groups compares the culture of *L. salivarius* ZJ614 and *L. reuteri* ZJ625 in defined media

For the secretome, the highest number of DEPs was observed between CLS vs CLRLS, followed by MLS vs MLSLR, and CLS vs MLS, while the lowest was observed between CLR vs CLRLS, followed by CLRLS vs MLRLS and CLR vs MLR. However, no DEPs were observed between MLR vs MLRLS. The observed DEPs include chaperonin, 50S ribosomal protein, cysteine-tRNA ligase, cell division protein, adenylosuccinate synthase, peptide chain release factor, phosphoglycerate kinase, glucose-6-phosphate isomerase, elongation factor, ribosome-recycling factor, chaperone protein, enolase, and S-adenosylmethionine, among others. Some important DEPs observed from the intracellularly and extracellularly expressed proteins, their quantitative profiles, and their biological functions are listed in Tables 2 and 3. The differential proteins are displayed as scatter and volcano plots (Supplementary Fig. 1), while the distribution is presented as a barplot (Fig. 2).

**Table 3:** Secretome: differentially expressed proteins between and within groups for the culture of *L. salivarius* ZJ614 and *L. reuteri* ZJ625 in defined media

**Groups**	**Proteins (genes)**	**Quantitative profile**		**p-value**	**Functions/associated pathways**
		**Up regulated**	**Down regulated**		
**CLR vs CLRLS**	33kDA Chaperonin (*hsl*O)	CLR-S	CLRLS-S	< 0.00010	Prevent the irreversible aggregation of thermally unfolding and oxidatively damaged proteins. Aid in bacterial defence response against oxidative stress
**CLR vs MLR**	50S ribosomal protein (*rpm*F)	CLR-S	MLR-S	< 0.00010	Ribosomal protein L32; Belongs to the bacterial ribosomal protein bL32 family
	Cysteine–tRNA ligase (cysS)	MLR-S	CLR-S	< 0.00010	Converts cysteine in the presence of ATP to AMP diphosphate, L-cysteinyl-tRNA (Cys)
	Cell division protein (*Fts*Z)	MLR-S	CLR-S	0.00025	it creates the Z ring at the site of cell division which form a septum between dividing cells. The assembly of Z rings determines the timing and position of cell division to attract other proteins involved in the process leading to the formation of a new cell wall that separates the two daughter cells
CLRLS vs MLSLR	Adenylosuccinate synthetase (*pur*A)	MLSLR-S	CLRLS-S	< 0.00010	Functions in the de novo biosynthesis of purine nucleotide and catalyses the biosynthesis of AMP from IMP
	Peptide chain release factor (*prf*A)	MLSLR-S	CLRLS-S	< 0.00010	Regulates the translation termination in the presence of the stop codons UAG and UAA
**CLS vs CLRLS**	Phosphoglycerate kinase (*pgk*)	CLRLS-S	CLS-S	0.00098	Carbohydrate metabolism; glycolysis; convert D-glyceraldehyde 3-phosphate to form pyruvate
	Glucose-6-phosphate isomerase (pgi)	CLRLS-S	CLS-S	0.0011	Enhances the reversible conversion of glucose-6-phosphate to fructose-6-phosphate
	Elongation factor G (*fus*A)	CLRLS-S	CLS-S	0.0039	Speed up the translocation of GTP-dependent ribosome during translation elongation
	Ribosome-recycling factor (*frr*)	CLRLS-S	CLS-S	0.00091	Releases ribosomes from mRNA at the stop of protein biosynthesis and increases translation efficiency by recycling ribosomes from one translation round to another
	Chaperone protein (DnaK)	CLS-S	CLRLS-S	0.0023	Acts as a chaperone; Belongs to the heat shock protein 70 family
	Ornithine carbamoyl transferase (*arc*B)	CLRLS-S	CLS-S	0.00083	Produces L-citrulline by facilitating the transfer of the carbamoyl group from carbamoyl phosphate to the N(epsilon) atom of ornithine
	Aspartyl/glutamyl-tRNA (Asn/Gln) amidotransferase subunit B	CLRLS-S	CLS-S	0.00044	Allows the formation of correctly charged Asn-tRNA (Asn) or Gln-tRNA (Gln) through the transamidation of misacylated Asp-tRNA (Asn) or Glu-tRNA (Gln) in organisms which lack either or both of asparaginyl-tRNA or glutaminyl-tRNA synthetases. The reaction takes place in the presence of glutamine and ATP through an activated phospho-Asp-tRNA (Asn) or phospho-Glu-tRNA (Gln)
	Enolase (*eno*)	CLS-S	CLRLS-S	0.0057	Catalyses the reversible conversion of 2-phosphoglycerate into phosphoenolpyruvate. It is essential for the degradation of carbohydrates via glycolysis
	S-adenosylmethionine synthase (*met*K)	CLS-S	CLRLS-S	0.0036	Catalyses the formation of S-adenosylmethionine (AdoMet) from methionine and ATP
	Arginine deiminase (*arc*A)	CLRLS-S	CLS-S	0.0015	Amino-acid degradation; L-arginine degradation via ADI pathway; carbamoyl phosphate from L-arginine
	PII-type proteinase (prtP)	CLS-S	CLRLS-S	0.00042	Protease which breaks down milk proteins during the growth of the bacteria on milk
	Phosphoglucosamine mutase (glmM)	CLS-S	CLRLS-S	0.0031	Catalyses the conversion of glucosamine-6-phosphate to glucosamine-1-phosphate
	Elongation factor P (efP)	CLRLS-S	CLS-S	0.0012	Protein biosynthesis; Involved in peptide bond synthesis. Stimulates efficient translation and peptide-bond synthesis on native or reconstituted 70S ribosomes in vitro. Probably functions indirectly by altering the affinity of the ribosome for aminoacyl-tRNA, thus increasing their reactivity as acceptors for peptidyl transferase
	Purine nucleoside phosphorylase (*deo*D)	CLRLS-S	CLS-S	0.0040	Catalyses the reversible phosphorolytic breakdown of the N-glycosidic bond in the beta-(deoxy) ribonucleoside molecules, with the formation of the corresponding free purine bases and pentose-1-phosphate
	Adenylate kinase (adk)	CLRLS-S	CLS-S	0.0016	Catalyses the reversible transfer of the terminal phosphate group between ATP and AMP. Plays an important role in cellular energy homeostasis and in adenine nucleotide metabolism
	Triosephosphate isomerase (tpiA)	CLS-S	CLRLS-S	0.00023	Gluconeogenesis: stereo specifically converts dihydroxyacetone phosphate (DHAP) to D-glyceraldehyde-3-phosphate
	Formate-tetrahydrofolate ligase (fhs)	CLRLS-S	CLS-S	0.00055	One-carbon metabolism; tetrahydrofolate interconversion
	Uridylate kinase (pyrH)	CLRLS-S	CLS-S	0.0014	Pyrimidine metabolism; CTP biosynthesis via de novo pathway; Catalyses the reversible phosphorylation of UMP to UDP
	Dipeptidase A (pepDA)	CLS-S	CLRLS-S	0.0057	Hydrolyses a wide range of dipeptides but unable to hydrolyse dipeptides containing proline
	S-ribosylhomocysteine lyase (luxS)	CLRLS-S	CLS-S	0.0019	Involved in the synthesis of autoinducer 2 (AI-2) which is secreted by bacteria and is used to communicate cell density and the metabolic potential of the environment. The regulation of gene expression in response to changes in cell density is called quorum sensing
	Galactose-1-phosphate uridylyltransferase (galT)	CLS-S	CLRLS-S	0.0015	Carbohydrate metabolism; galactose metabolism
	NH(3)-dependent NAD( +) synthetase (nadE)	CLRLS-S	CLS-S	< 0.00010	Catalyses the ATP-dependent amidation of deamido-NAD to form NAD. Uses ammonia as a nitrogen source
**CLS vs MLS**	Mannitol-1-phosphate 5-dehydrogenase (mltD)	CLS-S	MLS-S	< 0.00010	Involves in fructose and mannose metabolism
	6-phospho-beta-galactosidase (lacG)	CLS-S	MLS-S	0.00030	Carbohydrate metabolism; lactose degradation; D-galactose 6-phosphate and beta-D-glucose from lactose 6-phosphate
	Galactose-1-phosphate uridylyl transferase (galT)	CLS-S	MLS-S	0.00021	Carbohydrate metabolism; galactose metabolism
**MLS vs MLRLS**	Chaperone protein (DnaK)	MLSLR-S	MLS-S	0.00025	Acts as a chaperone
	Peptide chain release factor 1 (*prfC*)	MLSLR-S	MLS-S	< 0.00010	Increases the formation of ribosomal termination complexes and stimulates activities of RF-1 and RF-2. It binds guanine nucleotides and has strong preference for UGA stop codons. It may interact directly with the ribosome. The stimulation of RF-1 and RF-2 is significantly reduced by GTP and GDP, but not by GMP
	Galactokinase (galK)	MLSLR-S	MLS-S	< 0.00010	Catalyses the transfer of the gamma-phosphate of ATP to D-galactose to form alpha-D-galactose-1-phosphate (Gal-1-P)
	Thiamine-phosphate synthase (thiE)	MLSLR-S	MLS-S	0.00038	Condenses 4-methyl-5-(beta-hydroxyethyl) thiazole monophosphate (THZ-P) and 2-methyl-4-amino-5-hydroxymethyl pyrimidine pyrophosphate (HMP-PP) to form thiamine monophosphate (TMP)
	Ribosome maturation factor RimM (rim)	MLSLR-S	MLS-S	0.00028	An accessory protein needed during the final step in the assembly of 30S ribosomal subunit, possibly for assembly of the head region
	Peptide deformylase (def)	MLSLR-S	MLS-S	0.00028	Removes the formyl group from the N-terminal Met of newly synthesized proteins. Requires at least a dipeptide for an efficient rate of reaction. N-terminal L-methionine is a prerequisite for activity but the enzyme has broad specificity at other positions

### Functional Enrichment Analyses of the Differentially Expressed Proteins

**Fig. 3:**
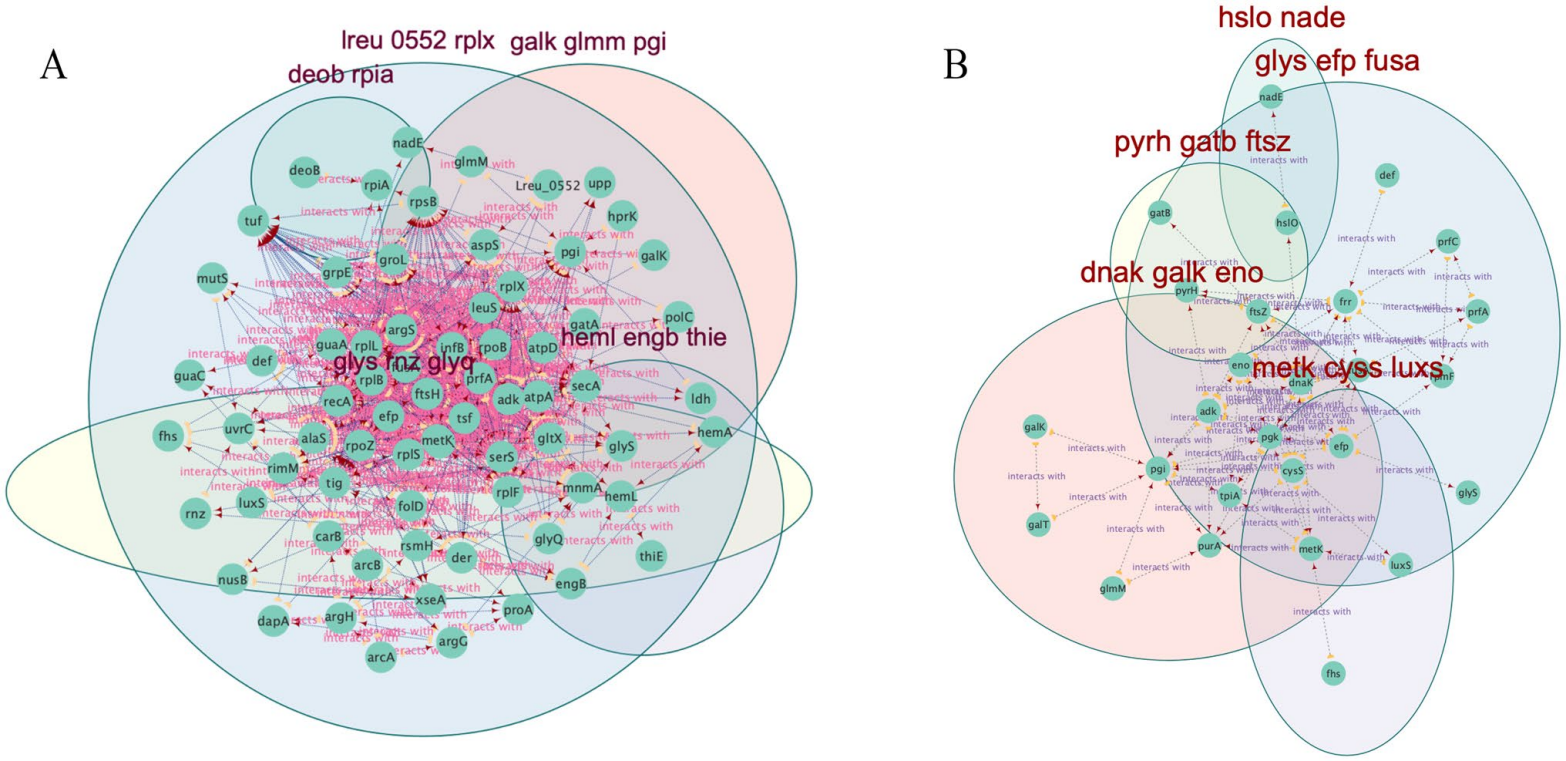
Protein-protein interactions network for the differentially expressed proteins from the **A** intracellular proteome and **B** extracellular (secretome) proteome. The arrows indicate the direction of the interactions with the heads denoting the target protein, while the yellow half circle the source of the interaction. The PPI networks are sub-clustered based on the biological relationships between the proteins

**Fig. 4:**
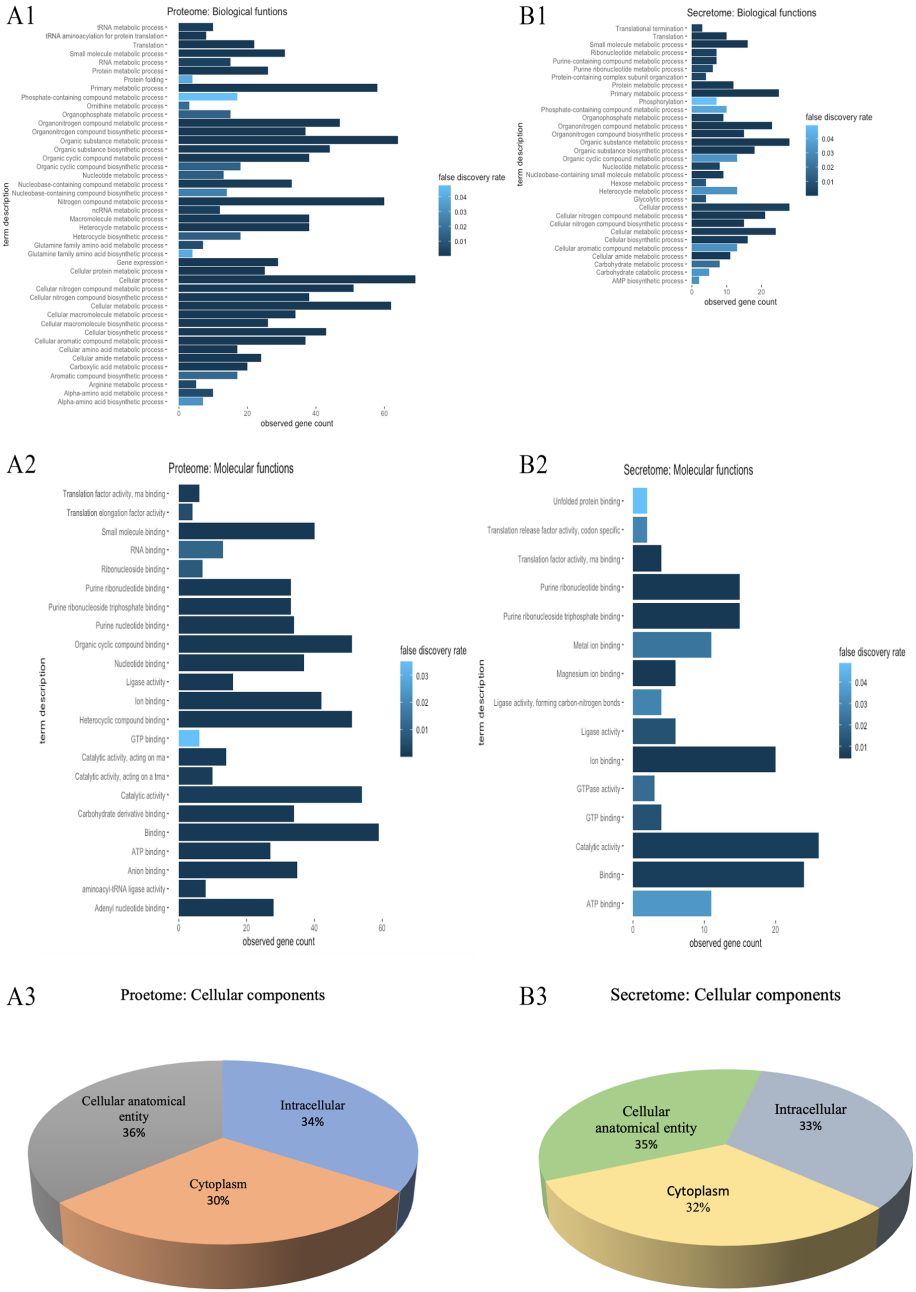
The enrichment and functional analyses of the differentially expressed proteins between *L. reuteri* ZJ625 and *L. salivarius* ZJ614 in defined media. (A1–A3) show the enrichment analyses of the intracellular proteome, and (B1–B3) are the enrichment and functional analyses of the DEPs from the extracellular proteome

**Fig. 5:**
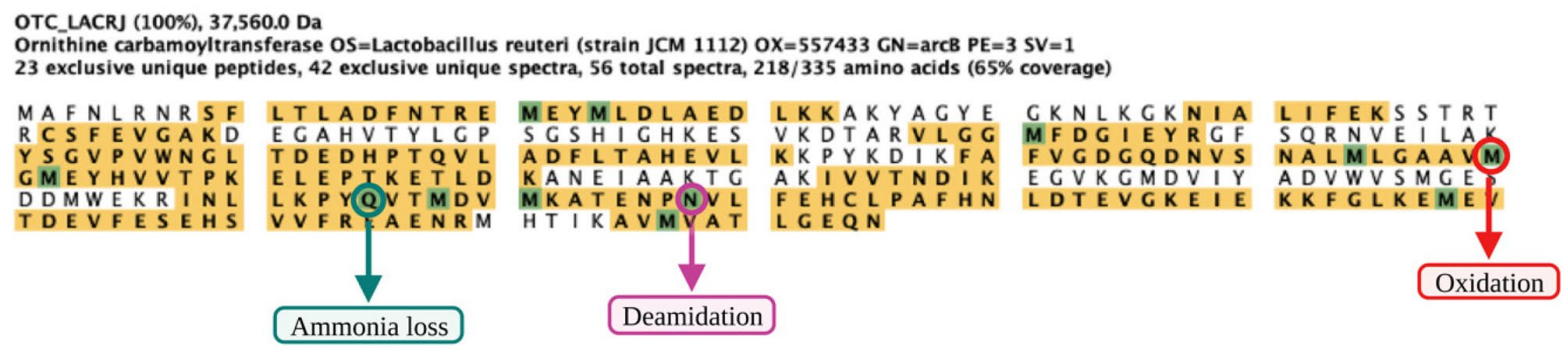
Post-translational modifications associated with the differentially expressed proteins from the growth of *L. salivarius* ZJ614 and *L. reuteri* ZJ625 in defined media

To map the metabolic potential of the differentially expressed proteins between and within the groups, we conducted network enrichment analyses against *L. reuteri* and *L. salivarius* genomes. Overall, the network analyses for both the intracellularly and extracellularly expressed proteomes indicated that the protein-protein interactions networks have significantly more interactions than expected (very low *p*-value < 0.005) (Fig. 3). Thus, revealing partial biological connections of the proteins as a group (Table 4). The DEPs are associated with several biological processes and molecular functions and are distributed in the bacteria’s cellular, anatomical entities, cytoplasm, and intracellular components (Fig. 4). The metabolic pathway enrichment analysis revealed the association of the DEPs in important metabolic pathways, including cellular metabolic processes, organic substances metabolism, protein metabolism, and transcription/translation processes. In the same way, metabolic functional analysis revealed the involvement of the DEPs in enzymatic activities, RNA binding, translation, and protein transport across the cell. (Fig. 5)

**Table 4:** Network stat analyses of the DEPs (genes) for the culture of *L. salivarius* ZJ614 and *L. reuteri* ZJ625 in defined media

**Parameters**	**Intracellular proteome**		**Extracellular proteome**	
	***L. salivarius***	***L. reuteri***	***L. salivarius***	***L. reuteri***
Number of nodes	71	72	28	30
Number of edges	776	518	119	71
Average node degree	21.9	14.4	8.5	4.73
Average local clustering coefficient	0.557	0.567	0.558	0.612
Expected number of edges	610	302	92	44
PPI enrichment *p*-value	5.89e-11	< 1.0e-16	0.00353	8.45e-05

### Post-Translation Modifications

Different post-translational modifications were identified on the proteins from the other experimental groups. The post-translational modifications associated with the significantly differential proteins include oxidation, deamidation, ammonia loss, methylthiolation, and dehydration. Table 5 lists examples of some of the DEPs and the associated PTMs. However, the same PTMs were observed in intracellular and extracellularly expressed proteomes, with variations in the associated experimental groups.

**Table 5:** Some examples of post-translational modification associated with some significantly differential protein

**Samples**	**Proteins**	**Post-translational modifications**
CLRLS	S-ribosylhomocysteine lyase (*lux*S)	Methylthio
CLRLS	Transcription antitermination	Deamidated
CLRLS, CLS	Ornithine carbamoyl transferase	Oxidation, deamidated, methylthio, dehydrated
CLRLS, CLS	Phosphoglycerate kinase	Oxidation, deamidated
CLR, MLR	ATP synthase subunit alpha	Oxidation, deamidated, dehydrated
CLRLS, MLRLS,	Protein translocase subunit SecA	Oxidation
CLRLS, MLRLS	Elongation factor TU (EF-TU)	Oxidation, deamidated
CLRLS, MLRLS	Chaperonin GroEL	Oxidation, ammonia-loss, deamidated
MLS, MLRLS	Aspartate—tRNA ligase	Oxidation, ammonia loss

## Discussion

The species of the genus *Lactobacillus* include several beneficial strains with roles in fermented food products and as probiotics, revealed by their genomes harbouring genes with functional traits [49–51]. *Limosilactobacillus reuteri* ZJ625 and *Ligilactobacillus salivarius* ZJ614 are important potential probiotics with benefits, including pathogenic microbe inhibition, suppression of anti-inflammatory cytokines, and improvement of gastrointestinal functions in post-weaning piglets through the modulation of the intestinal microbiota [24]. Dlamini et al. [24] showed that the functional effects of consuming *L. salivarius* ZJ614 and *L. reuteri* ZJ625 were enhanced in multi-strain preparation compared to their single-strain formulations. Previous studies have also revealed the preference for multi-strain probiotics over single-strain preparations due to the strain, disease, and host specificities of different probiotic microbes [52, 53]. However, the mechanisms of the enhanced benefits derived from multi-strain probiotics must be better understood. To elucidate the interactions between *L. salivarius* ZJ614 and *L. reuteri* ZJ625, we used labelled-free proteomics to study the mechanisms of cell-to-cell crosstalk between the two isolates in co-cultures using defined media. Defined media were used for culturing the isolates to avert the confounding challenges associated with data interpretation using undefined enriched media [40, 54, 55].

In this study, the intracellular and extracellular proteomes from the different experimental groups were compared to determine the variations in the protein abundance in each group. Although there are variations in the amount and identities of the proteins expressed by the strains in the experimental groups, the DEPs are similar and largely exhibit very close biological and molecular functions. Overall, higher amounts of proteins and DEPs were observed from the intracellular proteome than the extracellular proteome. This variation could be because proteins are synthesized intracellularly, and most of the cell’s metabolic activities occur intracellularly [56]. The variations in the protein abundance between and within groups indicate the influence of the different culture conditions and especially the absence of some media components, including some minerals (cobalt (II) chloride, calcium chloride, zinc sulphate, potassium chloride, and copper sulphate), amino acids (L-alanine, L-valine, L-proline, and L-aspartic acid), nucleotide bases (guanine, adenine, xanthine, and nicotinic acid), and vitamins (thiamine hydrochloride, folic acid, p-aminobenzoic acid, and biotin) in the minimal defined medium.

Functional annotation of the differential proteins revealed their roles in several biological and molecular processes. For example, the expression of 50S ribosomal protein that maintains the structure and function of the aminoacyl-tRNA binding site during translation [57] was up-regulated in the co-culture in CDM (CLRLS) compared to the single culture of *L. reuteri* ZJ625 (CLR). However, the differential expressions of most of the proteins between CLR and CLRLS were insignificant, indicating a less effect of co-culturing on the proteome of *L. reuteri* ZJ625 in the presence of *L. salivarius* ZJ614 in CDM. In contrast, comparing the proteins expressed between the co-culture (CLRLS) and CLS (*L. salivarius* ZJ614) showed higher significantly expressed differential proteins between the two groups. The expression of elongation factor G, a protein involved in accurate protein synthesis under stress conditions [58], was higher in the CLRLS than in CLS. Similarly, some important proteins, such as enolase and phosphoglycerate kinase, necessary for glucose degradation during glycolysis and pyruvate synthesis from D-glyceraldehyde 3-phosphate [58], were highly expressed in CLRLS compared to CLS. The genes that code for these proteins were previously reported as adaptational genes acquired by LAB for survival in nutrient-rich growth medium [59]. These findings indicate the effect of medium components and co-culturing on the intracellular and extracellularly expressed proteome of *L. salivarius* ZJ614 and *L. reuteri* ZJ625, denoting possible synergy, additive, and/or inhibitory interactions between the two strains. Interestingly, these results indicate the roles of the differential proteins in converting glucose during glycolysis, pentose phosphate pathway, and pyruvate production by the co-culture. This was evidenced by the high levels of phosphoglycerate kinase and enolase that catalyse the production of phosphoenolpyruvate from 2-phosphoglycerate. However, the expression of L-lactate dehydrogenase that breaks fructose to pyruvate [60] was highly expressed in CLS compared to CLRLS, denoting the ability of *L. reuteri* ZJ625 to influence the fermentation by *L. salivarius* ZJ614 in a mixed-culture. Between-group comparison of *L. salivarius* ZJ614 on MDM and CDM indicates no significant differential proteins signifying the ability of *L. salivarius* ZJ614 to thrive and maintain metabolic activities despite the absence of some medium components. Also, no significant differential proteins were observed between MLR and MLRLS, similar to what was observed between CLR and CLRLS.

Between groups, comparisons also revealed the high expression of serine t-ligase that binds serine to tRNA in CLR compared to MLR. Enolase, an enzyme that enzymatically converts phosphoglycerate into phosphoenolpyruvate in a reversible reaction, was highly expressed in CLR compared to MLR. However, the ATP synthase subunit-α which synthesises ATP from ADP across the membrane was highly expressed in MLR compared to CLR. Also, a high level of ornithine carbamoyl transferase transfers the carbamoyl group to the N-atom of ornithine during the L-citrulline synthesis [58] was observed in MLR compared to CLR. The significant difference in the proteome of *L. reuteri* ZJ625, when cultured in different media (either MDM or CDM), indicates the response of the strain to changes in growth media components. Similar to the proteome of *L. reuteri* ZJ625 in a minimal medium, the co-culture also showed high expression of translocase subunit *Sec*A, which catalyses ATP binding and import across the cell membrane into the cell when compared to CLRLS. Another protein highly expressed in MLRLS compared to CLRLS is GMP synthase, which is involved in glutamine biosynthesis. However, the trigger factor that acts as a chaperone in protein folding and maintaining freshly produced proteins in opened conformation was highly expressed in CLRLS, unlike MLRLS. Comparison of the isolates on the MDM revealed aspartate tRNA ligase, which aids in nucleic acid binding and aspartyl-tRNA aminoacylation. However, no significant DEPs were found between MLR and MLRLS. Even though several differential proteins were observed between CLS and MLS, no significant differences were found in their expression levels.

Quorum sensing is a bacterial cell-to-cell chemical communication in which bacteria respond to population density gradient through the production, detection, and response to extracellular signalling molecules known as autoinducers [61]. Bacteria integrate the information encoded through quorum sensing for within-species, within-genera, and between-species crosstalk, as well as interactions with the gut microbiome [62]. In this study, S-ribosylhomocysteine lyas (*lux*S) was significantly high in the co-culture from the minimal medium (MDM) compared to the same in the complete defined medium (CDM). This protein synthesises autoinducer-2 (AI-2), used to communicate the cell density and the metabolic potential of the environment between bacteria. It also regulates the transformation of S-ribosylhomocysteine to homocysteine and 4,5-dihydroxy-2,3-pentadione [63]. Autoinducer-2 is associated with probiotics interaction with the microbiome and stimulates the proliferation of Firmicutes during antimicrobial-associated dysbiosis [64]. The high expression of *lux*S by the strains co-cultured in MDM indicates the mechanisms of intercellular crosstalk between *L. salivarius* ZJ614 and *L. reuteri* ZJ625 and the potential ability of the strains to modulate the gut microbiome while check-mating their population density. The presence of several important proteins, such as elongation factor TU, glyceraldehyde-3-phosphate dehydrogenase, and the phosphor-carrier protein (HPr), previously reported to play roles in Lactobacilli attachment to the host epithelium [65], indicates the potential for crosstalk between the isolates in this study during probiotic-host interactions. Glyceraldehyde-3-phosphate dehydrogenase also possesses antigenic and immunomodulatory effects, stimulates B and T-cell activation, and increases IL-10 secretion in the host [66]. However, since this study was conducted in vitro, this conclusion needs to be validated using biological systems (cell/tissue culture, animal model experiment).

The protein-protein interactions (PPI) network analyses for the intracellularly and extracellularly expressed proteomes indicate significantly more interactions than expected. The PPI indicate the involvement of the proteins in cell processes, including transcription/translation, protein transport, and nucleotide metabolism. The PPI further indicates the interactions of the quorum-sensing enzyme *lux*S with S-adenosylmethionine synthase (*met*k) and cysteine—tRNA ligase (*cys*S), which are important enzymes that play roles in methionine and cysteine biosynthesis, respectively. The interactions of *lux*S with *rpl*S, a protein sub-unit located on the 50S ribosomal protein and with roles in aminoacyl-tRNA binding, further indicate the role of the quorum-sensing enzyme in protein biosynthesis, as previously reported [67, 68].

Another important mechanism by which the living cells expand their chemical structure and information carried by the 20 proteinogenic amino acids is by binding covalent modifications to polypeptide chains. Most of these modifications are linked after synthesising the polypeptide chains (translation) and are termed post-translational modifications (PTMs) [69]. In this study, the post-translational modifications identified are oxidation, deamidation, methylthiolation, ammonia loss, and dehydration (Table 5). These PTMs were observed on proteins associated with different physiological processes, including transcription, amino acid degradation, glucose metabolism, ATP synthesis, and S-ribosylhomocysteine lyase (*lux*S) (involved in synthesising autoinducer-2). Proteins PTMs affect the chemical structures of the modified residues and closely associated polypeptide regions, influencing the protein conformation, folding, binding abilities, and, importantly, their functions [69]. For instance, thiol-based PTMs on S-ribosylhomocysteine lyase (*lux*S) and ornithine carbamoyltransferase were previously reported to induce several transcription factors involved in detoxification pathways [70] and redox regulation of cellular metabolism in bacteria [71]. Deamidation and ammonia loss involve the release of ammonia into the surrounding medium and function in the competitive survival strategy for available amino acids by lactic acid bacteria [72]. Bacteria employ post-translational modifications as crucial strategies to survive and adapt to their environment. PTMs control several aspects of protein functions and microbial physiology, such as protein-protein interactions, protein turnover, cell-to-cell crosstalk, and cell differentiation [73].

## Conclusion

The study elucidates the interactions and influence of co-culturing between *L. reuteri* ZJ625 and *L. salivarius* ZJ614 in defined media through label-free proteomics analysis. Co-culturing of the two strains resulted in the expression of differential proteins with biological, molecular, and enzymatic activities in the defined media. The differentially expressed proteins were associated with post-translational modifications such as deamidation, ammonia loss, oxidation, dehydration, and methylthiolation. Importantly, this study revealed the interactions between *L. reuteri* ZJ625 and *L. salivarius* ZJ614 mediated by the secretion of S-ribosylhomocysteine lyase (*lux*S) useful in an autoinducer-2 synthesis useful in cell-to-cell bacterial crosstalk in co-cultures. The proteome of *L. salivarius* ZJ614 was most affected when co-cultured with *L. reuteri* ZJ625. In contrast, the effect of removing medium components from the minimal medium was more evident on *L. reuteri* ZJ625 than on *L. salivarius* ZJ614. To fully explore the benefits of multi-strain probiotics for biological applications, ex vivo and in vivo proteomics studies should be carried out. This will grant the understanding of the effects of probiotics in the host and the mechanisms of probiotics-microbiome modulation.

## Electronic Supplementary Material

Below is the link to the electronic supplementary material.

Supplementary Material 1 (PDF 729 KB)

## Data Availability

The data from this study will be made available by the corresponding author upon reasonable request.
