# Supplementary material for: Elucidating the Mechanisms of Cell-to-Cell Crosstalk in Probiotics Co-culture: A Proteomics Study of Limosilactobacillus reuteri ZJ625 and Ligilactobacillus salivarius ZJ614
Source: Probiotics Antimicrob Proteins. 2023 Aug 15;16(5):1817–35. doi: 10.1007/s12602-023-10133-y (PMC11445297; doi:10.1007/s12602-023-10133-y)
Supplement: Supplementary file 1 — Supplementary Material 1 (PDF 729 KB) [file 12602_2023_10133_MOESM1_ESM.pdf]

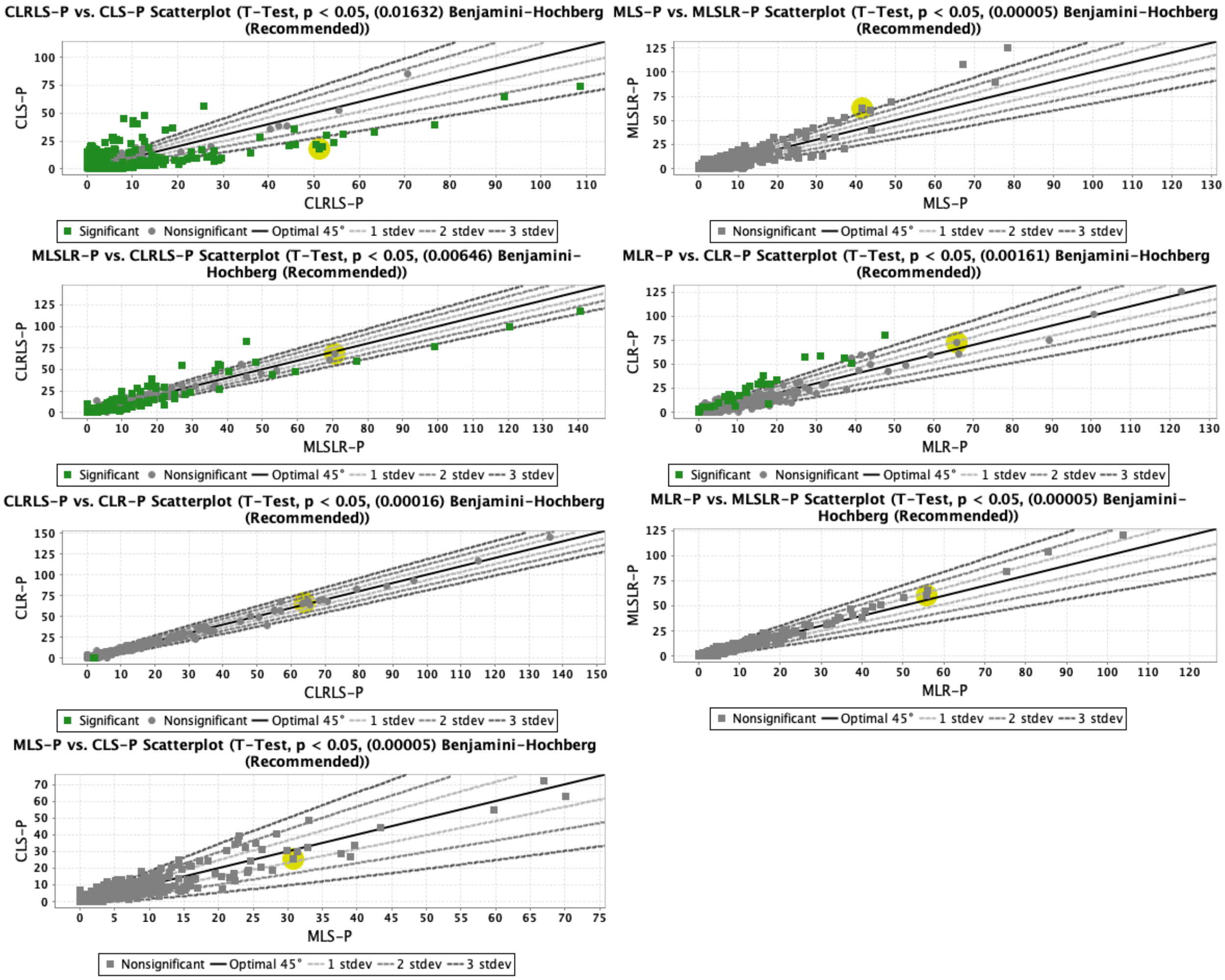

Supplementary Figure 1A: scatter plots (intracellular proteins)

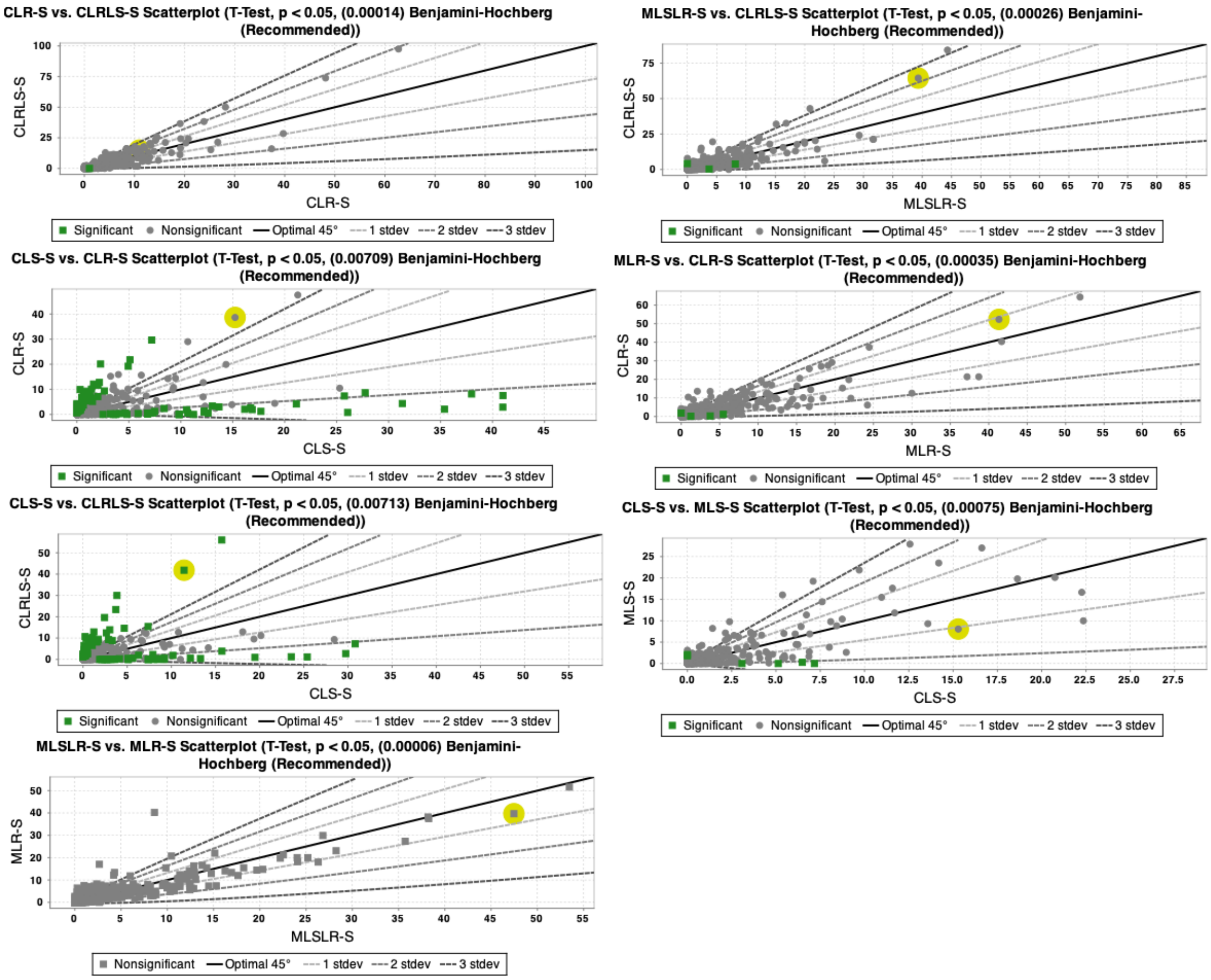

Supplementary Figure 1B: Scatter plots (Extracellular proteome)

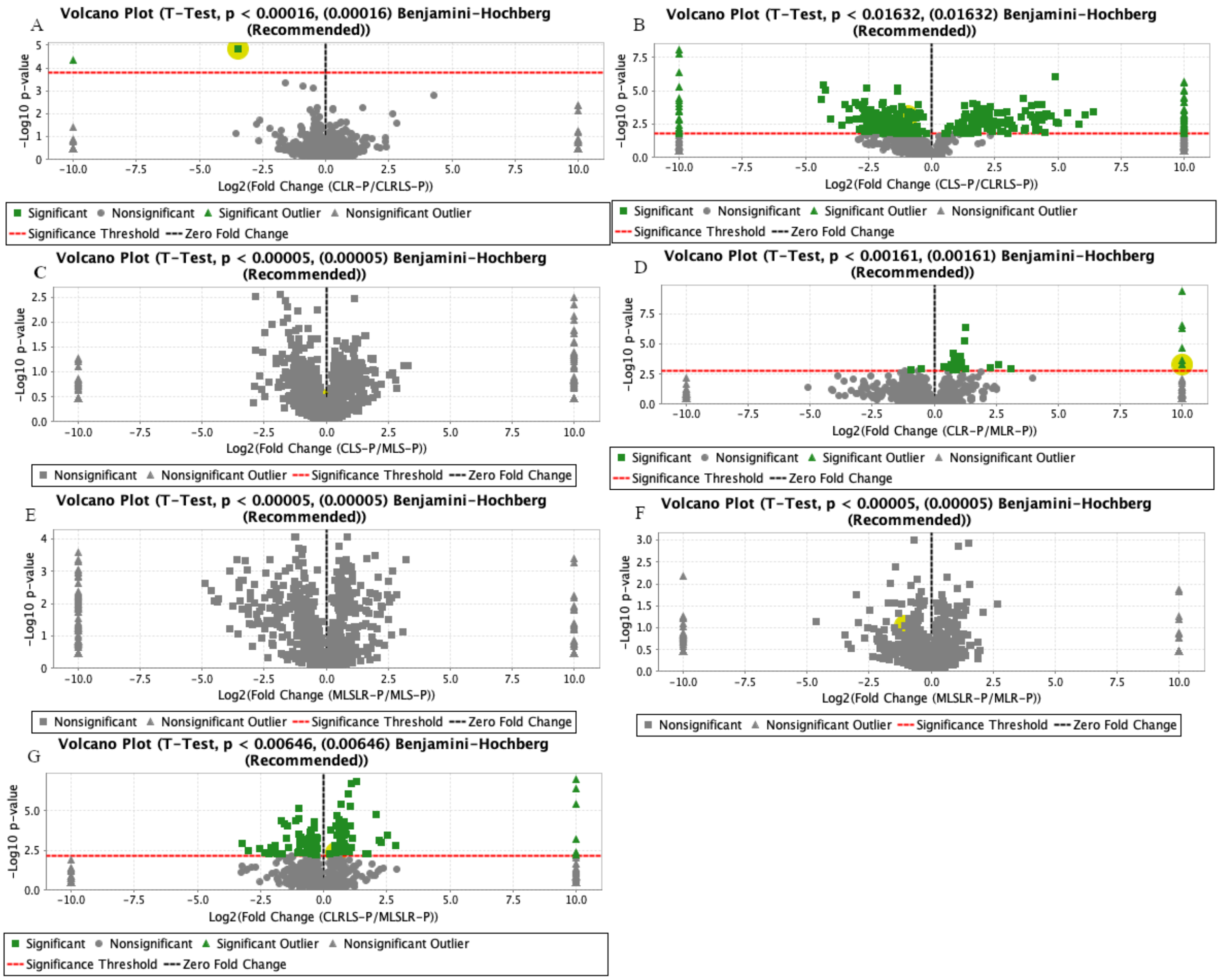

Supplementary Figure 1C: Volcano plots (intracellular proteome)

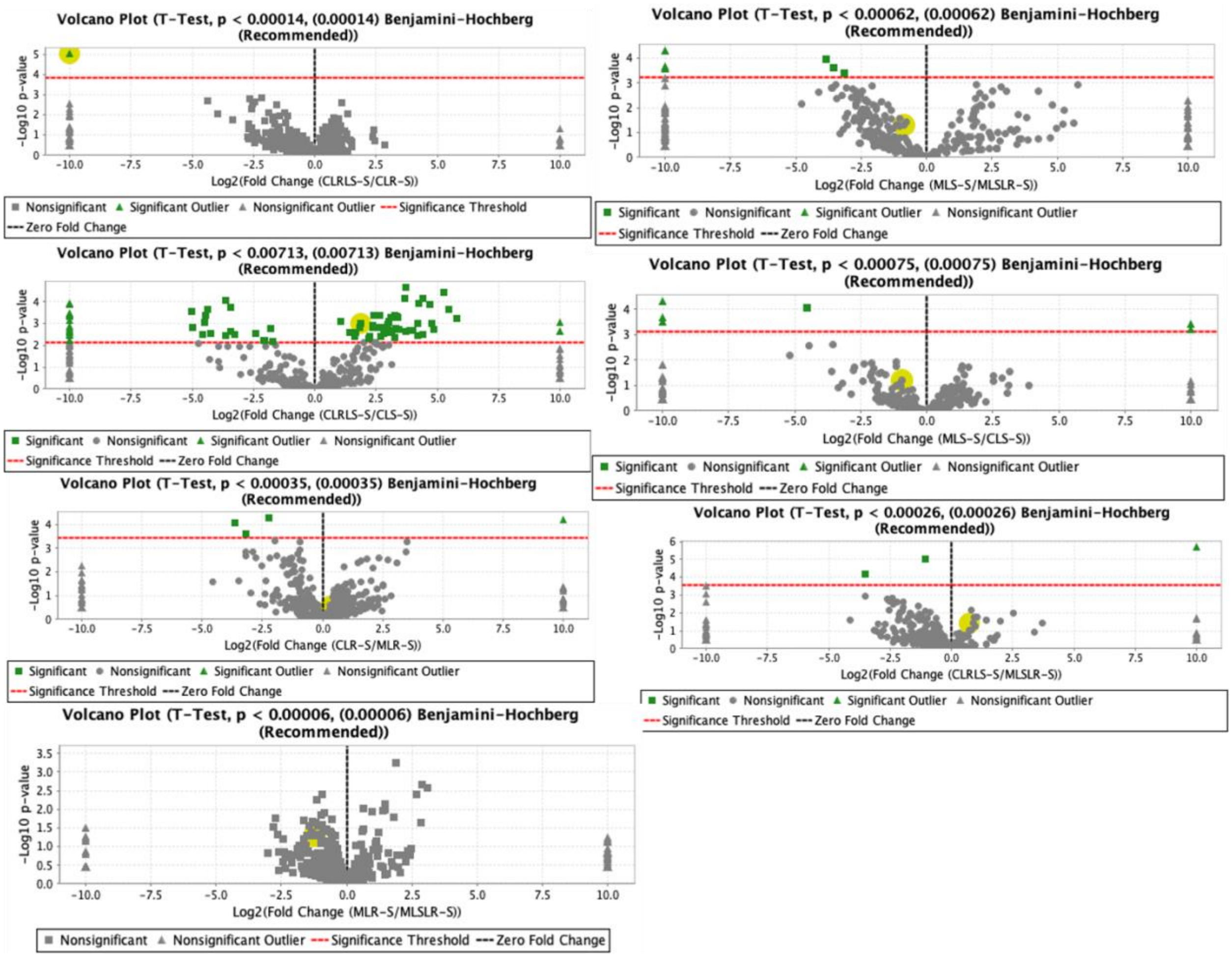

Supplementary Figure 1D: Volcano plots (Extracellular proteome)
